# Feathered Detectives: Real-Time GPS Tracking of Scavenging Gulls Pinpoints Illegal Waste Dumping

**DOI:** 10.1371/journal.pone.0159974

**Published:** 2016-07-22

**Authors:** Joan Navarro, David Grémillet, Isabel Afán, Francisco Ramírez, Willem Bouten, Manuela G. Forero

**Affiliations:** 1 Estación Biológica de Doñana CSIC, Avda. Américo Vespucio s/n, Sevilla, 41092, Spain; 2 Centre d’Ecologie Fonctionnelle et Evolutive, UMR 5175, CNRS - Université de Montpellier - Université Paul-Valéry Montpellier - EPHE, Montpellier, France; 3 Percy Fitz Patrick Institute, DST/NRF Centre of Excellence, University of Cape Town, Cape Town, South Africa; 4 Computational Geo-Ecology, Institute for Biodiversity and Ecosystem Dynamics (IBED), University of Amsterdam, 1090 GE, Amsterdam, The Netherlands; INIBIOMA (Universidad Nacional del Comahue-CONICET), ARGENTINA

## Abstract

Urban waste impacts human and environmental health, and waste management has become one of the major challenges of humanity. Concurrently with new directives due to manage this human by-product, illegal dumping has become one of the most lucrative activities of organized crime. Beyond economic fraud, illegal waste disposal strongly enhances uncontrolled dissemination of human pathogens, pollutants and invasive species. Here, we demonstrate the potential of novel real-time GPS tracking of scavenging species to detect environmental crime. Specifically, we were able to detect illegal activities at an officially closed dump, which was visited recurrently by 5 of 19 GPS-tracked yellow-legged gulls (*Larus michahellis*). In comparison with conventional land-based surveys, GPS tracking allows a much wider and cost-efficient spatiotemporal coverage, even of the most hazardous sites, while GPS data accessibility through the internet enables rapid intervention. Our results suggest that multi-species guilds of feathered detectives equipped with GPS and cameras could help fight illegal dumping at continental scales. We encourage further experimental studies, to infer waste detection thresholds in gulls and other scavenging species exploiting human waste dumps.

## Introduction

The world’s human population produces > 3 million tonnes of solid waste per day, more than all other anthropogenic emissions, including greenhouse gases [1,2]. Waste impacts on human health and the environment are severe, creating a hugely topical issue across societies. This is the case within the European Union, where the Landfill Directive (Council Directive 99/31/EC) aims at reducing, recycling, composting, landfilling or incinerating human waste, to mitigate its deleterious impacts. However, these measures are costly, and the immense market generated by the waste business has led to a surge in illegal activities [3,4]. Specifically, illegal trafficking of human waste has recently become one of the most lucrative activities of organized crime [3]. This is famously the case in southern Italy, where the mafia colludes with local institutions to control waste markets, with consequences for the environment and people’s health [5,6]. This type of environmental crime has also been recently reported in other European countries such as Spain, Greece, France, Romania, Bulgaria and the United Kingdom, based on infringement proceedings opened by the Court of Justice of the European Union (http://europa.eu/about-eu/institutions-bodies/court-justice/index_en.htm), by further official reports, and by the media.

Beyond economic fraud, these illegal activities strongly enhance the uncontrolled dissemination of human pathogens, pollutants and invasive species [7–9]. Governments are therefore putting particular emphasis on detecting and restricting illegal dumping activities [10], but they are faced with extremely volatile practices, resulting in more or less unpredictable occurrence of illegal waste [11,12]. Here, we demonstrate the potential of novel real-time GPS tracking of scavenging species for rapidly and effectively detecting illegal urban dumps. In particular, we investigate the spatial movements of yellow-legged gulls (*Larus michahellis*), a model species of scavenging gull keen on human organic waste [13]. We thereby show how GPS-tracked gulls pinpoint illegal waste dump activity in southern Spain, a region where this activity is a standing practice [10].

## Material and Methods

### Ethics statement

All fieldwork was reviewed and specifically approved by the Ethics Committee of CSIC (REF: 28-04-15-237), in accordance with the Spanish and EU legislation on the protection of animals used for scientific purposes. Permits to work in the natural protected Biosphere Reserve of Marismas de Odiel were provided by the Junta de Andalucía (Spain). The field study did not involve endangered or protected species.

### Fieldwork procedures

**Fig 1:**
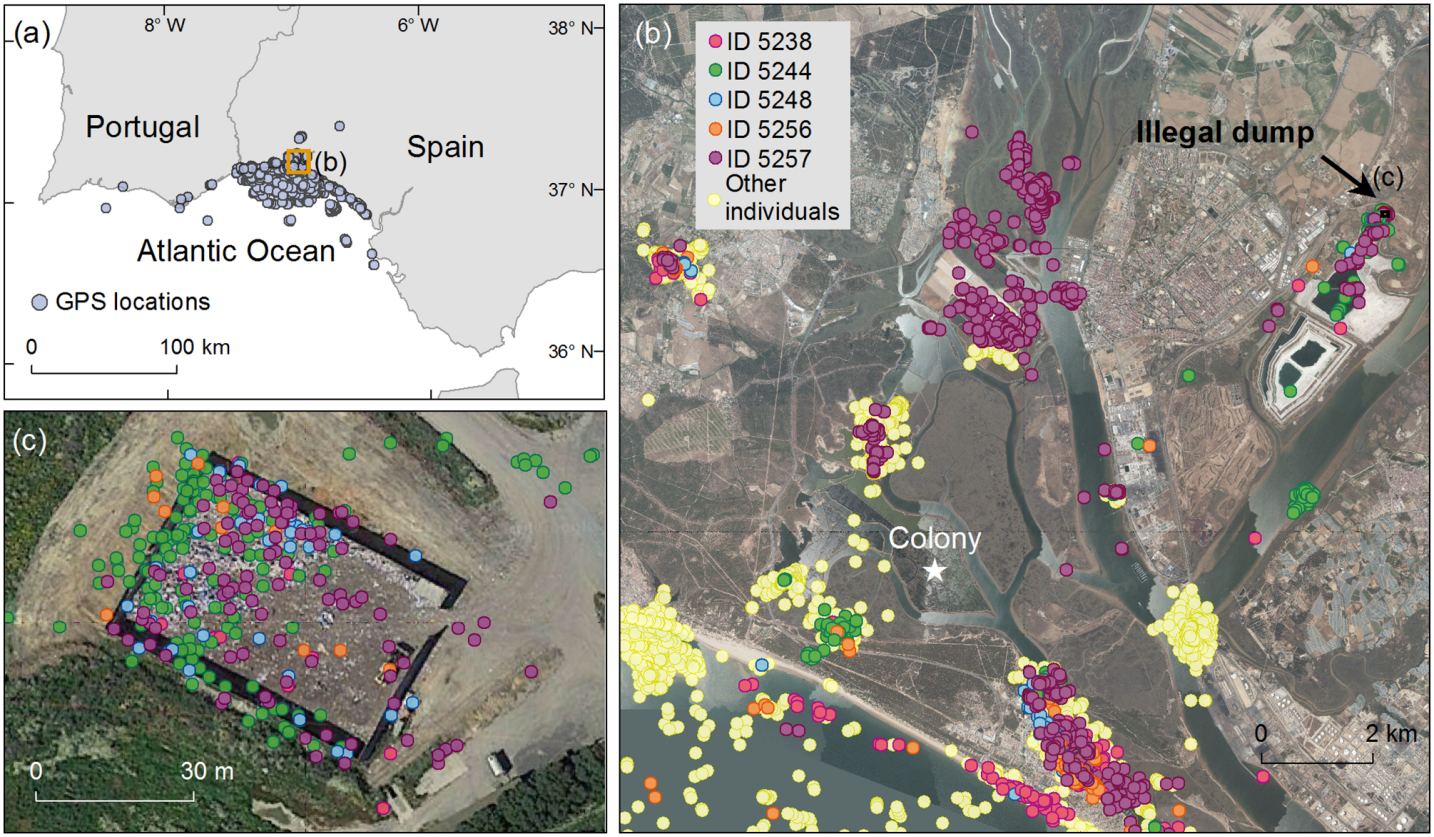
Spatial distribution of 19 yellow-legged gulls equipped with GPS loggers between 7-May-2015 and 15-June-2015 while breeding at the natural protected Biosphere Reserve of Marismas de Odiel (southwestern Iberian Peninsula, Spain). (a) overall distribution, (b) zoom onto a particular area showing different individuals, and (c) zoom onto the illegal urban waste dump used recurrently by five individuals. The map is made by ArcGIS 10.3 software (ESRI; academic licences provided by CSIC). Aerial images were obtained during the PNOA programme (National Programme of Aerial Orthography, Ministry of Development, http://www.pnoa.ign.es). Images were freely provided by the IGN (Spanish National Geographic Institute, http://www.ign.es/ign). Spatial scale was 1:2500 and last update was from year 2011.

From 7^th^ May to 15^th^ June 2015, coinciding with their breeding period, we investigated the spatial movements of 19 yellow-legged gulls breeding at the natural protected Biosphere Reserve of Marismas de Odiel (southwestern Iberian Peninsula, Spain; Fig 1). Adult birds were equipped with solar-powered GPS trackers (http://www.UvA-BiTS.nl; University of Amsterdam, The Netherlands, [14]) recording their location every 5 minutes during the same period. Uva-BiTS loggers can recharge themselves using solar energy, allowing to track the movements of birds continuously during several years [14]. We caught the gulls at the nest during the incubation using a walk-in wire mesh trap. GPS loggers were attached using wing harnesses constructed of tubular Teflon^™^ ribbon (Bally Ribbon Mills 8475- .25”). The combined weight of the GPS devices plus harness was less than the 2.5% of the body mass of the bird. GPS data were automatically downloaded remotely from devices to a field-based laptop when the birds were present at the breeding colony, where a network of 3 antennas provided complete coverage of the breeding area [14]. GPS data was parsed into the central database and immediately available in the UvA-BiTS Virtual Lab for visualization and data exploration [14].

### Data analysis

All GPS positions within the colony or categorized as travelling locations were discarded, and we only locations associated with active foraging (<5 m·s^-1^) were kept. We subsequently qualified habitat use by overlapping filtered foraging locations with high-resolution land cover information (SIOSE, Soil Information System of Spain, Junta de Andalucía, last update 2011) and geographical references of regulated waste dumps (Spatial Reference Databases of Andalucía, DERA, last update 21/02/2014). This classification was reviewed carefully with the most recent satellite image offered by Google Earth V 7.1.2.2041 (19/4/2013) at 0.5 m spatial resolution, which allowed the visual observation of open-air dump activities. GPS foraging locations were finally classified into five habitat categories: marine (sea and fishing ports), estuarine, freshwater (water ponds, fish farms and wetlands), terrestrial (agriculture, urban areas and beach), and waste dumps. Crucially, for those waste dumps we could distinguish legal, operational sites, from those which had previously been closed, and had therefore become illegal.

## Results

**Fig 2:**
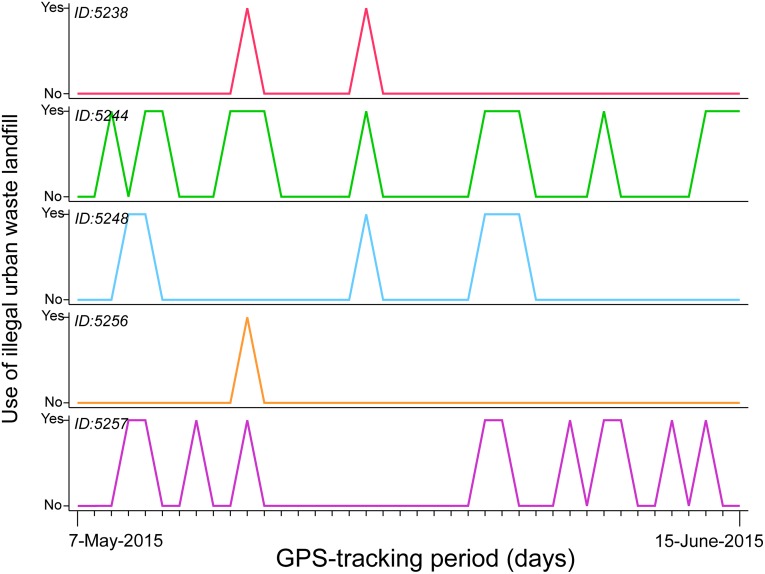
Activity of 5 yellow-legged gulls equipped with GPS loggers on the illegal dump. The data was obtained between 7-May-2015 and 15-June-2015 at the natural protected Biosphere Reserve of Marismas de Odiel (southwestern Iberian Peninsula, Spain).

GPS data (20,497 locations; 1,078±350 locations·individual^-1^) revealed that the 19 tracked yellow-legged gulls patrolled a vast area (9,444 km^2^) with a maximum range of 122 km from the breeding colony, visiting Spain and Portugal (Fig 1A). Regarding the habitat used during foraging activities, we found that tracked gulls mainly exploited the marine environment (44.4% of total GPS positions), followed by estuaries (21.6%), freshwater habitats (10.7%), terrestrial habitats (16.0%), saltpans (6.2%) and waste dumps (1.1%) (Fig 1A). The latter habitat category includes two different waste dumps, one of them illegal (Fig 1C). It is important to specify that as we started analysing the data, we did not know about this illegal dump, and it is really visits by gulls to this particular site which attracted our attention. This small illegal dump (of an extension of 0.001 km^2^) that occupied <1% of the total gull homerange was located 10 km away from the breeding colony, and was used repeatedly during different days by five birds (25% of the 19 individuals) during the study period (Fig 2). Specifically, 18% of all locations recorded for these five individuals were within this illegal dump site (Fig 1C).

## Discussion

Our data demonstrate that scavenging gulls equipped with real-time GPS recorders can provide accurate and immediate information about the existence and location of illegal urban waste dumps. Gulls were able to clearly pinpoint a waste dump which was officially closed after the European landfill regulation ~10 years ago. Gull foraging activity clearly revealed that this regulation is not being implemented. Scavengers such as the yellow-legged gull actively search for any organic matter, and are well known for their ability to exploit human organic waste [13]; before European landfill regulations, the population trends of these opportunistic gulls were often directly linked to waste availability [15]. However, one may wonder why only 5 of the 19 gulls which we GPS-tracked exploited urban waste dumps. This result may be related to the low availability of urban dumps (legal and potentially illegal ones) in the area patrolled by GPS-tracked individuals. Based on official data, only two regulated dumps are in the area, one of them was visited by one GPS-tracked individual and we suspect (based on the tracking data and information provided by the staff of the Natural Park) that only the illegal dump detected by the gulls was active during the study period. Visual inspections confirmed that yellow-legged gulls and others scavengers such as white storks (*Ciconia ciconia*) visited this dump and were looking for food among urban garbage site for feeding (Navarro, personal observation). In addition to other prey present in the dumps such insects and small mammals, scavenger birds mainly used dumps to consume the organic matter present in human waste [13,16,17]. Therefore, potentially if there had been a greater number of legal/illegal dumps accessible for the gulls (open area and without bird barriers) in the study area, more birds would have visited them. In our case, 25% of all the birds were able to detect this illegal activity, and then 18% of all locations recorded for these birds pinpointed the illegal dump. Hence, equipping a reasonable number of birds will ensure that any illegal dumping activity containing organic refuses will be detected, and this is why we argue that feathered detectives may provide a substantial aid to waste management.

In this context, GPS tracking has major advantages when compared with other methodologies used to detect illegal dumping, such as land-based monitoring of dumps or scavenger aggregations, the use of aerial photographs, or of predictive models based on spatial data (see Table 1; [10,18,19]. Firstly, gulls allow a much wider and cost-efficient spatial cover than conventional land-based surveys. Crucially, birds completely ignore borders, personal and air-traffic regulations, and leisurely fly into areas which would be extremely hazardous for the general public, or even for law-enforcement. Secondly, the GPS tracking system was developed such that the data is almost immediately available, at a high spatio-temporal resolution. In our study, for instance, we could not only detect the illegal activity of a dumping site, but also determine where and when organic waste was being dumped, with unprecedented spatiotemporal resolution. Also, although we did not provide non-breeding information, the GPS units we used, and other remote-GPS loggers, record and transmit the same data outside the breeding season. From an economic point of view, the use of GPS tracking is clearly less expensive than other methods such as drones’ censuses. For example, the average cost of the 19 loggers used in the present study was 30 euros·h^-1^ (cost of the 19 loggers = 19,000 euros, cost of the reception system = 9,000 euros, working time = 39 days sampling rate 5 minutes), several orders of magnitude less than the cost of conducting similar work using a drone that ranges between 500–1,000 euros·h^-1^ (total cost for 39 days of a drone census of 46,800–93,600 euros, according to quotations provided by companies which operate drones and associated pilots). Since illegal dumping activities are very irregular in time (not daily) and place (usually dumpers try to avoid reusing the same place), to fly a drone only one day in a particular area does not seem very efficient. For this reason we considered the entire GPS-tracing period across which the gulls were patrolling.

**Table 1:** Examples of advantages and limitations of some methods used to detect illegal dumping.

Method	Advantages	Limitations
GPS-loggers (with remote data-download capability)	Cost-efficient; Real-time information; 24-hours of data; Large patrolled area; No legal limitations in the movements; Non-human dependent	Need to capture free-living birds; Illegal dumps need to be inside the foraging area covered by the GPS-tracked species; Only useful if the dump contains organic matter
GPS-loggers (without remote download capability)	Cost-efficient; 24-hours of data; Large patrolled area; No limitations in the movements; Non-human dependent	Need to capture and recapture free-living birds; Some days of delay in the spatial information; Illegal dumps need to be inside the foraging area covered by the GPS-tracked species; Only useful if the dump contains organic matter
Unnamed Aerial Systems/ Drones	Real-time information; Visual information; Useful to detect all type of illegal dump	High expensive; Legal restrictions in the access to private areas or to cross country borders; Small patrolled area; Short-time autonomy
Land-based monitoring	Real-time information; Visual information; Useful to detect all type of illegal dump	Legal restrictions in the access to private areas or to cross country borders; Small patrolled area; High effort to cover wide areas
Aerial photographs	Visual information; High extension covered; Useful to detect all type of illegal dump	Non-real time information; moderately costly; High time to cover wide areas

**Fig 3:**
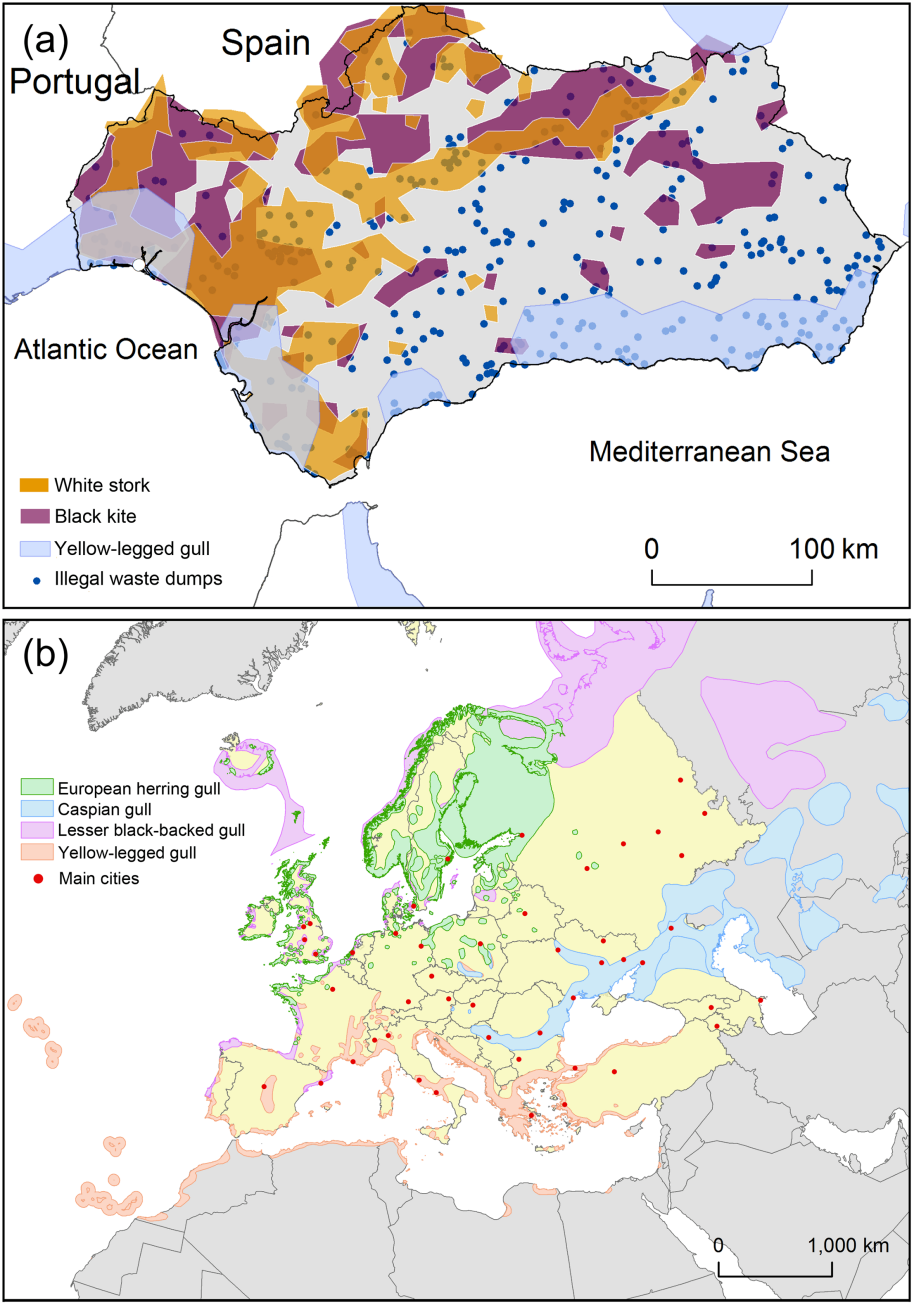
A network of feathered detectives. (a) Spatial distribution of reported illegal human waste dumps (blue dots; obtained from [10]) and three scavenger birds (yellow-legged gull *Larus michaellis*, white stork *Ciconia ciconia* and black kite *Milvus migrans*) in the southern Spain, and (b) overall spatial distribution during breeding and wintering periods of four scavenger gull species (European herring gull *Larus argentatus*, Caspian gull *Larus cachinnans*, lesser black-backed gull *Larus fuscus* and yellow-legged gull) and the main cities (red points; population greater than one million inhabitants) across western Europe (in yellow). Spatial distribution (colour contours) was provided by BirdLife International and NatureServe (http://www.birdlife.org) and National Inventory of Biodiversity, Ministry of Agriculture, Food and Environment, Spanish Government. The map is made by ArcGIS 10.3 software (ESRI).

One limitation of the ‘feathered detective approach’ is that it only allows the detection of illegal waste which contains organic matter, to the exclusion of other type of human waste such construction, electronic or hazardous inert waste. Further, one may argue that a specific bird species might be constrained, in time and space. To counter this, a multi-species guild of feathered detectives might also be used for the detection of environmental crime at larger spatio-temporal scales. For example, in southern Spain, there is a strong spatial overlap between the distribution of three scavengers which exploit human waste at dumps (yellow-legged gull, white stork, and black kite *Milvus migrans*) [17,20], and of potentially illegal waste dumps [10] (see Fig 3B). Also, the possibility to use trained scavengers to detect urban waste has been reported recently in Peru (http://www.gallinazoavisa.pe/).

Yet, the use of feathered detectives may also create collateral casualties, as persons engaged in illegal waste dumping may try to shoot them or to put poisoned baits once they suspect that they may record sensitive information. This possibility cannot be completely excluded, yet, past experience has shown that it is not that easy to kill wild birds so as to eradicate a whole group, unless very substantial efforts are invested, which may make criminals extremely conspicuous to the public, and to law enforcement. Specifically, real-time online information about feathered detectives may substantially raise the awareness of the public with respect to illegal waste disposal (see example in http://www.gallinazoavisa.pe/), and make illegal waste dumping far less efficient. Yet, it is clear that only abundant species, whose populations are not diminished by the impacts of anthropogenic threats, should be used as feathered detectives.

In conclusion, GPS-tracking of scavenging birds may be an additional, surprisingly efficient tool to fight illegal waste dumping which contains organic garbage. Animal-borne cameras, used in combination with GPS-trackers, will soon allow a better characterization of waste contents [21,22], similar to their utility to address conservation and management issues in oceanic environments [23]. Moreover, the widespread distributions during the breeding and wintering periods of different scavenger gull species across Europe (Fig 3B), which overlaps with some of the most important European cities, suggest a potential use of this methodology at a continental scale. We also encourage additional experimental studies to examine their utility during the non-breeding season, and to examine the usefulness of feathered detectives to detect illegal dumping events occurring at other places than closed waste dumps, and to infer waste detection thresholds (time/ distance of detection or the minimum waste size to be detected) in gulls and other scavenging species.
